# 5,6,7,8-Tetra­hydro­quinoline 1-oxide hemihydrate

**DOI:** 10.1107/S1600536810008779

**Published:** 2010-03-13

**Authors:** Zbigniew Karczmarzyk, Teodozja M. Lipińska, Waldemar Wysocki, Zofia Urbańczyk-Lipkowska, Przemysław Kalicki

**Affiliations:** a Department of Chemistry, University of Podlasie, ul. 3 Maja 54, 08-110 Siedlce, Poland; b Institute of Organic Chemistry, Polish Academy of Sciences, ul. Kasprzaka 44/52, 01-224 Warsaw 42, POB 58, Poland

## Abstract

In the title compound, C_9_H_11_NO·0.5H_2_O, the asymmetric unit contains two similar mol­ecules of 5,6,7,8-tetra­hydro­quinoline 1-oxide and one water mol­ecule. The water mol­ecule links the two O atoms of both independent *N*-oxides into dimers *via* O—H⋯O hydrogen bonds, forming a three-dimensional network along [101], which is additionally stabilized by weak C—H⋯O inter­molecular inter­actions. In each mol­ecule, the saturated six-membered rings exist in a conformation inter­mediate between a half-chair and sofa.

## Related literature

For background to the chemistry of the title compound and its applications, see: Coperet *et al.* (1998 ▶); Li (2005 ▶); Kaiser *et al.* (2006 ▶); Kaczorowski *et al.* (2009 ▶). For the synthesis, see: Jacobs *et al.* (2000 ▶); Barbay *et al.* (2008 ▶). For the biological activity of 5,6,7,8-tetra­hydro­quinoline derivatives, see: Calhoun *et al.* (1995 ▶); Abd El-Salam *et al.* (2009 ▶). For a related structure, see: HXTHQO (CSD, November 2009 release). For structure inter­pretation tools, see: Duax & Norton (1975 ▶); Allen *et al.* (1987 ▶); Allen (2002 ▶); Bruno *et al.* (2002 ▶).
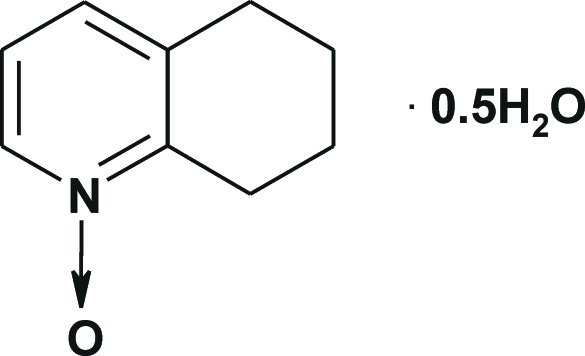


## Experimental

### 

#### Crystal data

C_9_H_11_NO·0.5H_2_O
*M* *_r_* = 158.20
Orthorhombic, 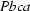

*a* = 14.725 (4) Å
*b* = 14.464 (4) Å
*c* = 15.474 (3) Å
*V* = 3295.7 (14) Å^3^
*Z* = 16
Cu *K*α radiation
μ = 0.70 mm^−1^
*T* = 293 K
0.28 × 0.26 × 0.21 mm

#### Data collection

Bruker SMART APEXII CCD diffractometer
Absorption correction: multi-scan (*SADABS*; Bruker, 2005 ▶) *T*_min_ = 0.832, *T*_max_ = 0.873
11258 measured reflections
2727 independent reflections
1989 reflections with *I* > 2σ(*I*)
*R*_int_ = 0.053

#### Refinement

*R*[*F* ^2^ > 2σ(*F* ^2^)] = 0.051
*wR*(*F* ^2^) = 0.205
*S* = 1.39
2727 reflections
215 parameters
H atoms treated by a mixture of independent and constrained refinement
Δρ_max_ = 0.44 e Å^−3^
Δρ_min_ = −0.24 e Å^−3^

### 

Data collection: *APEX2* (Bruker, 2005 ▶); cell refinement: *SAINT* (Bruker, 2005 ▶); data reduction: *SAINT*; program(s) used to solve structure: *SHELXS97* (Sheldrick, 2008 ▶); program(s) used to refine structure: *SHELXL97* (Sheldrick, 2008 ▶); molecular graphics: *ORTEP-3 for Windows* (Farrugia, 1997 ▶); software used to prepare material for publication: *SHELXL97* and *WinGX* (Farrugia, 1999 ▶).

## Supplementary Material

Crystal structure: contains datablocks I, global. DOI: 10.1107/S1600536810008779/jj2025sup1.cif

Structure factors: contains datablocks I. DOI: 10.1107/S1600536810008779/jj2025Isup2.hkl

Additional supplementary materials:  crystallographic information; 3D view; checkCIF report

## Figures and Tables

**Table 1:** Hydrogen-bond geometry (Å, °)

*D*—H⋯*A*	*D*—H	H⋯*A*	*D*⋯*A*	*D*—H⋯*A*
O2—H21⋯O1*A*	0.96 (3)	1.87 (3)	2.825 (3)	170 (3)
O2—H22⋯O1*B*	0.95 (4)	1.86 (3)	2.799 (3)	170 (3)
C2*B*—H2*B*⋯O1*A*	0.93	2.53	3.454 (3)	171
C3*A*—H3*A*⋯O2^i^	0.93	2.50	3.392 (3)	160
C3*B*—H3*B*⋯O2^ii^	0.93	2.56	3.342 (4)	142
C5*A*—H52*A*⋯O1*B*^iii^	0.97	2.49	3.383 (4)	153

Symmetry codes: (i) 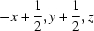; (ii) 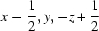; (iii) 
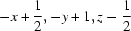
.

**Fig. 1.**
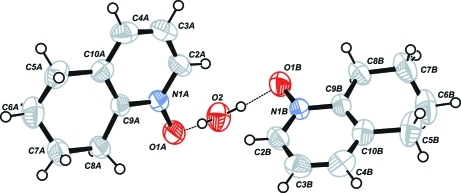
The molecular structure of (I), with atom labels and 50% probability displacement ellipsoids for non-H atoms. Dashed lines indicate O—H ··· O hydrogen bonds.

**Fig. 2.**
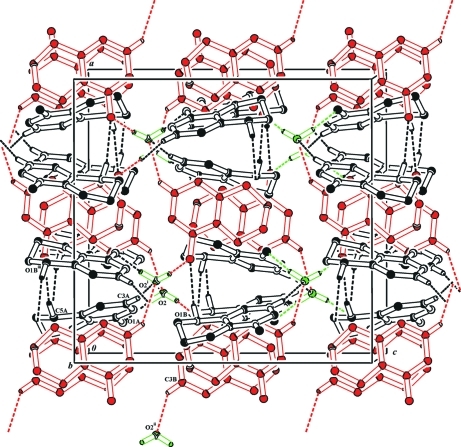
A view of the molecular packing in (I) (black - molecules A, red - molecules B, green - H2O). Dashed lines indicate O—H ··· O hydrogen bonds and weak C—H···O intermolecular interactions.

## supplementary crystallographic information

### Comment

5,6,7,8-Tetrahydroquinoline 1-oxide, (I), is an important intermediate for the synthesis of quinoline derivatives *via* Boekelheide rearrangement (Li, 2002; Kaiser *et al.*, 2006; Coperet *et al.*, 1998). The 5,6,7,8-tetrahydroquinoline moiety is found as a subunit in numerous medicinally interesting compounds (Calhoun *et al.*, 1995; Abd El-Salam *et al.*, 2009). Compound (I) can be obtained by the reaction of 5,6,7,8-tetrahydroquinoline with hydrogen peroxide or with MCPBA (Jacobs *et al.*, 2000; Barbay *et al.*, 2008). A search of the Cambridge Structural Database (November 2009 Release; Allen, 2002; Bruno *et al.*, 2002) showed 26 organic compounds with the 5,6,7,8-tetrahydroquinoline moiety. Due to our interest in the preparartion of new nanomaterials based on organometallic complexes similar to those obtained from Cinchona alkaloids (Kaczorowski *et al.*, 2009), a new method of synthesis for (I), by the oxidation of 5,6,7,8-tetrahydroquinoline with the catalytic system of oxone/TlOAc/PhI in a water-acetonitrile solution at room temperature has been developed and its crystal and molecular structure reported.

The asymmetric unit contains two similar molecules of 5,6,7,8-tetrahydroquinoline 1-oxide and one water molecule (Fig. 1). The water molecule links the two O atoms of both independent N-oxides by O—H···O hydrogen bonds into dimmers, which form a three-dimensional network along the [101] (Fig. 2). Additional weak C—H···O intermolecular interactions help stabilize the crystal packing (Table 1). The water molecule is observed in the ^1^H NMR spectrum as a broad signal at 2.4 ppm and in the IR spectrum as two absorption maxima for two different O—H bonds at 3368 and 3312 cm^-1^, respectively. The bond distances and angles in (I) are in normal ranges (Allen *et al.*, 1987) and are comparable to the corresponding values observed in related structure of 5-hydroxy-5,6,7,8-tetrahydroquinoline 1-oxide (HXTHQO; CSD, November 2009 Release). In (I) the 6-membered fused-ring systems of the molecules A and B, are observed in an intermediate conformation between a half-chair and sofa with asymmetry parameters Δ*C*_s_(C6A) = 13.4 (3)°, Δ*C*_2_(C6A,C7A) = 11.2 (4)°, Δ*C*_s_(C6B) = 11.1 (2)° and Δ*C*_2_(C6B, C7B) =14.8 (3)° (Duax & Norton, 1975).

### Experimental

The title compound, C_9_H_11_NO.0.5H_2_O, was synthesized by the oxidation process of the 5,6,7,8-tetrahydroquinoline with an oxone/TlOAc/PhI in water-acetonitrile solution, catalytic system at room temperature. To a solution of 5,6,7,8-tetrahydroquinoline (333 mg, 0.325 ml, 2.5 mmol,), in acetonitrile (7.5 ml) and water (7.5 ml), PhI (1.25 ml of a 0.1M solution in MeCN, 0.124 mmol) and thallous acetate (50 µl of 0.16M solution in water, 0.008 mmol) were added. Next, oxone (6.98 g, 11.5 mmol) was added in five portions over 6 h under stirring at room temperature. Substrate disappearing and new product forming was observed by TLC (*R*_f_ = 0.75 and *R*_f_ = 015, respectively, in ethyl acetate/methanol 50:1). The next day (after 20 h), 10% sodium hydroxide (10 ml), dichloromethane (30 ml) and water (30 ml) were added and the mixture was stirred for 5 min. The organic solution was separated and the aqueous phase was extracted with CH_2_Cl_2_ (2 x 15 ml). The combined organic phase was dried (anhydrous Na_2_SO_4_) and concentrated. Pure products 350 mg (95.0%) were obtained in oily form. After purification on column chromatography with silica gel and using ethyl acetate, the trace of the substrate was first removed. The product was eluted with a mixture of ethyl acetate/methanol (50:1) and colourless crystals were obtained. Yield: 310 mg (83%) and m.p. 344 K. Crystals suitable for X-ray diffraction analysis were grown by slow evaporation of a dichloromethane/hexane (1:10) solution.

### Refinement

The H atoms of the water molecule involved in the intramolecular hydrogen bonds were located by difference Fourier synthesis and refined freely [O—H = 0.96 (3) and 0.95 (4) Å]. The remaining H atoms were positioned geometrically and treated as riding on their C atoms, with C—H distances of 0.93 Å (aromatic) and 0.97 Å (CH_2_). All H atoms were refined with *U*_iso_(H) = 1.5*U*_eq_(O, C)].

### Crystal data

**Table:**

C_9_H_11_NO·0.5H_2_O	*F*(000) = 1360
*M**_r_* = 158.20	*D*_x_ = 1.275 Mg m^−^^3^
Orthorhombic, *P**b**c**a*	Cu *K*α radiation, λ = 1.54178 Å
Hall symbol: -P 2ac 2ab	Cell parameters from 3676 reflections
*a* = 14.725 (4) Å	θ = 5.7–66.9°
*b* = 14.464 (4) Å	µ = 0.70 mm^−^^1^
*c* = 15.474 (3) Å	*T* = 293 K
*V* = 3295.7 (14) Å^3^	Block, colourless
*Z* = 16	0.28 × 0.26 × 0.21 mm

### Data collection

**Table:**

Bruker SMART APEXII CCD diffractometer	2727 independent reflections
Radiation source: fine-focus sealed tube	1989 reflections with *I* > 2σ(*I*)
graphite	*R*_int_ = 0.053
φ and ω scans	θ_max_ = 65.4°, θ_min_ = 5.2°
Absorption correction: multi-scan (*SADABS*; Bruker, 2005)	*h* = −17→16
*T*_min_ = 0.832, *T*_max_ = 0.873	*k* = −16→17
11258 measured reflections	*l* = −18→10

### Refinement

**Table:**

Refinement on *F*^2^	Secondary atom site location: difference Fourier map
Least-squares matrix: full	Hydrogen site location: inferred from neighbouring sites
*R*[*F*^2^ > 2σ(*F*^2^)] = 0.051	H atoms treated by a mixture of independent and constrained refinement
*wR*(*F*^2^) = 0.205	*w* = 1/[σ^2^(*F*_o_^2^) + (0.1*P*)^2^] where *P* = (*F*_o_^2^ + 2*F*_c_^2^)/3
*S* = 1.39	(Δ/σ)_max_ < 0.001
2727 reflections	Δρ_max_ = 0.44 e Å^−^^3^
215 parameters	Δρ_min_ = −0.24 e Å^−^^3^
0 restraints	Extinction correction: *SHELXL97* (Sheldrick, 2008), Fc^*^=kFc[1+0.001xFc^2^λ^3^/sin(2θ)]^-1/4^
Primary atom site location: structure-invariant direct methods	Extinction coefficient: 0.0014 (4)

### Special details

**Table:**

Experimental. ^1^H MNR (400 MHz, CDCl_3_) *δ*: 8.13 (*d*, 1H, *J *= 6.0 Hz), 7.04–6.99 (*m*, 2H), 2.93 (*t*, 2H, *J* = 6.4 Hz), 2.75 (*t*, 2H, *J* = 6.4 Hz), 2.40 (*br s*, 1H), 1.92–1.85 (*m*, 2H), 1,78–1.72 (*m*, 2H); ^13^C MNR (100 MHz, CDCl_3_) *δ*: 148.8, 136.9, 136.4, 126.6, 121.9, 28.6, 24.6, 21.8, 21.6; IR (KBr, *ν*, cm^-1^): 3368 (*s*, OH), 3312 (*s*, OH), 3076 (*m*), 3050 (*m*), 3009 (*m*), 2935 (*s*), 2871 (*m*), 2837 (*m*), 2498 (*w*), 2410 (*w*), 2151 (*w*), 1970 (*w*), 1686 (*m*, NO), 1596 (*m*), 1482 (*m*), 1449 (*s*), 1334 (*m*), 1253 (*s*), 1232 (*s*), 1211 (*s*), 1194 (*s*), 1155 (*m*), 1089 (*m*), 1074 (*s*), 1041 (*m*), 971 (*s*), 897 (*m*), 865 (*w*), 830 (*m*), 797 (*s*), 701 (*m*), 676 (*m*).
Geometry. All esds (except the esd in the dihedral angle between two l.s. planes) are estimated using the full covariance matrix. The cell esds are taken into account individually in the estimation of esds in distances, angles and torsion angles; correlations between esds in cell parameters are only used when they are defined by crystal symmetry. An approximate (isotropic) treatment of cell esds is used for estimating esds involving l.s. planes.
Refinement. Refinement of *F*^2^ against ALL reflections. The weighted *R*-factor wR and goodness of fit *S* are based on *F*^2^, conventional *R*-factors *R* are based on *F*, with *F* set to zero for negative *F*^2^. The threshold expression of *F*^2^ > σ(*F*^2^) is used only for calculating *R*-factors(gt) etc. and is not relevant to the choice of reflections for refinement. *R*-factors based on *F*^2^ are statistically about twice as large as those based on *F*, and *R*- factors based on ALL data will be even larger.

### Fractional atomic coordinates and isotropic or equivalent isotropic displacement parameters (Å^2^)

**Table:**

	*x*	*y*	*z*	*U*_iso_*/*U*_eq_	
O1A	0.11925 (15)	0.33158 (10)	0.13008 (9)	0.0703 (6)	
N1A	0.13994 (15)	0.41708 (12)	0.11018 (11)	0.0502 (6)	
C2A	0.16947 (18)	0.47498 (16)	0.17206 (15)	0.0581 (7)	
H2A	0.1737	0.4547	0.2290	0.087*	
C3A	0.1930 (2)	0.56261 (17)	0.15166 (17)	0.0666 (8)	
H3A	0.2143	0.6025	0.1942	0.100*	
C4A	0.1853 (2)	0.59244 (16)	0.06758 (18)	0.0669 (8)	
H4A	0.2018	0.6527	0.0536	0.100*	
C5A	0.1449 (2)	0.56465 (16)	−0.08980 (17)	0.0686 (8)	
H51A	0.0900	0.6010	−0.0964	0.103*	
H52A	0.1961	0.6041	−0.1041	0.103*	
C6A	0.1422 (2)	0.4849 (2)	−0.15194 (16)	0.0774 (9)	
H61A	0.1268	0.5075	−0.2091	0.116*	
H62A	0.2018	0.4566	−0.1551	0.116*	
C7A	0.0749 (2)	0.41457 (18)	−0.12508 (14)	0.0673 (8)	
H71A	0.0741	0.3649	−0.1672	0.101*	
H72A	0.0149	0.4424	−0.1242	0.101*	
C8A	0.09560 (19)	0.37485 (14)	−0.03650 (13)	0.0534 (7)	
H81A	0.0408	0.3472	−0.0132	0.080*	
H82A	0.1405	0.3262	−0.0426	0.080*	
C9A	0.13012 (16)	0.44502 (14)	0.02589 (12)	0.0455 (6)	
C10A	0.15331 (17)	0.53397 (15)	0.00343 (15)	0.0520 (6)	
O1B	0.13846 (14)	0.36329 (13)	0.38241 (10)	0.0701 (6)	
N1B	0.05059 (15)	0.35441 (11)	0.39183 (10)	0.0489 (6)	
C2B	−0.0012 (2)	0.33685 (15)	0.32153 (14)	0.0571 (7)	
H2B	0.0258	0.3314	0.2674	0.086*	
C3B	−0.0921 (2)	0.32725 (16)	0.32982 (17)	0.0647 (8)	
H3B	−0.1277	0.3148	0.2815	0.097*	
C4B	−0.1320 (2)	0.33585 (16)	0.40978 (19)	0.0658 (7)	
H4B	−0.1946	0.3297	0.4153	0.099*	
C5B	−0.1198 (3)	0.3615 (2)	0.57197 (19)	0.0843 (11)	
H51B	−0.1695	0.3179	0.5776	0.126*	
H52B	−0.1441	0.4232	0.5800	0.126*	
C6B	−0.0486 (3)	0.3418 (2)	0.64194 (17)	0.0941 (12)	
H61B	−0.0747	0.3538	0.6984	0.141*	
H62B	−0.0315	0.2771	0.6397	0.141*	
C7B	0.0323 (3)	0.3992 (2)	0.63022 (15)	0.0853 (11)	
H71B	0.0747	0.3870	0.6768	0.128*	
H72B	0.0151	0.4638	0.6331	0.128*	
C8B	0.0784 (2)	0.38036 (16)	0.54433 (14)	0.0591 (7)	
H81B	0.1162	0.4329	0.5295	0.089*	
H82B	0.1177	0.3270	0.5505	0.089*	
C9B	0.01368 (19)	0.36319 (13)	0.47254 (13)	0.0475 (6)	
C10B	−0.0792 (2)	0.35369 (15)	0.48235 (15)	0.0569 (7)	
O2	0.21964 (17)	0.24427 (13)	0.26255 (13)	0.0781 (7)	
H21	0.188 (2)	0.269 (2)	0.213 (2)	0.117*	
H22	0.195 (2)	0.280 (2)	0.308 (2)	0.117*	

### Atomic displacement parameters (Å^2^)

**Table:**

	*U*^11^	*U*^22^	*U*^33^	*U*^12^	*U*^13^	*U*^23^
O1A	0.1126 (19)	0.0511 (9)	0.0473 (9)	−0.0176 (9)	−0.0026 (9)	0.0080 (7)
N1A	0.0606 (16)	0.0485 (10)	0.0415 (9)	−0.0037 (8)	0.0024 (8)	−0.0043 (8)
C2A	0.0615 (18)	0.0618 (14)	0.0510 (11)	−0.0026 (12)	−0.0041 (11)	−0.0143 (11)
C3A	0.067 (2)	0.0616 (14)	0.0708 (15)	−0.0049 (12)	−0.0088 (14)	−0.0202 (12)
C4A	0.070 (2)	0.0476 (12)	0.0833 (17)	−0.0082 (12)	0.0021 (15)	−0.0046 (12)
C5A	0.079 (2)	0.0593 (14)	0.0676 (14)	−0.0017 (13)	0.0070 (14)	0.0177 (12)
C6A	0.099 (3)	0.0823 (18)	0.0509 (12)	0.0069 (16)	0.0040 (14)	0.0130 (13)
C7A	0.086 (3)	0.0698 (16)	0.0463 (12)	0.0007 (14)	−0.0053 (12)	−0.0003 (11)
C8A	0.069 (2)	0.0491 (12)	0.0426 (11)	−0.0037 (10)	0.0005 (11)	−0.0051 (9)
C9A	0.0486 (16)	0.0471 (11)	0.0408 (10)	0.0002 (9)	0.0054 (10)	−0.0019 (9)
C10A	0.0497 (17)	0.0473 (12)	0.0590 (12)	−0.0006 (9)	0.0060 (11)	0.0004 (11)
O1B	0.0602 (16)	0.0916 (13)	0.0584 (10)	−0.0088 (10)	0.0101 (9)	−0.0125 (9)
N1B	0.0553 (16)	0.0495 (10)	0.0418 (9)	−0.0027 (8)	0.0034 (9)	−0.0025 (8)
C2B	0.071 (2)	0.0567 (13)	0.0439 (11)	−0.0008 (12)	−0.0046 (12)	−0.0036 (10)
C3B	0.074 (2)	0.0562 (14)	0.0638 (15)	−0.0020 (12)	−0.0162 (14)	0.0025 (11)
C4B	0.0531 (19)	0.0587 (14)	0.0855 (18)	0.0083 (12)	0.0009 (15)	0.0121 (13)
C5B	0.089 (3)	0.0870 (19)	0.0769 (18)	0.0294 (17)	0.0379 (18)	0.0192 (15)
C6B	0.142 (4)	0.086 (2)	0.0537 (15)	0.026 (2)	0.0270 (18)	0.0116 (14)
C7B	0.136 (4)	0.0739 (17)	0.0454 (13)	0.0120 (19)	0.0020 (16)	−0.0034 (13)
C8B	0.081 (2)	0.0520 (12)	0.0446 (11)	0.0045 (11)	−0.0058 (12)	−0.0046 (10)
C9B	0.0632 (19)	0.0377 (10)	0.0416 (11)	0.0064 (9)	0.0042 (10)	0.0016 (8)
C10B	0.065 (2)	0.0469 (12)	0.0583 (13)	0.0125 (11)	0.0090 (12)	0.0092 (10)
O2	0.0836 (19)	0.0752 (12)	0.0755 (11)	0.0184 (10)	0.0147 (11)	0.0021 (9)

### Geometric parameters (Å, °)

**Table:**

O1A—N1A	1.310 (2)	N1B—C2B	1.353 (3)
N1A—C2A	1.344 (3)	N1B—C9B	1.368 (3)
N1A—C9A	1.373 (3)	C2B—C3B	1.352 (4)
C2A—C3A	1.351 (3)	C2B—H2B	0.9300
C2A—H2A	0.9300	C3B—C4B	1.375 (4)
C3A—C4A	1.375 (4)	C3B—H3B	0.9300
C3A—H3A	0.9300	C4B—C10B	1.390 (4)
C4A—C10A	1.387 (4)	C4B—H4B	0.9300
C4A—H4A	0.9300	C5B—C10B	1.514 (4)
C5A—C6A	1.503 (4)	C5B—C6B	1.534 (5)
C5A—C10A	1.514 (3)	C5B—H51B	0.9700
C5A—H51A	0.9700	C5B—H52B	0.9700
C5A—H52A	0.9700	C6B—C7B	1.462 (5)
C6A—C7A	1.480 (4)	C6B—H61B	0.9700
C6A—H61A	0.9700	C6B—H62B	0.9700
C6A—H62A	0.9700	C7B—C8B	1.517 (4)
C7A—C8A	1.517 (3)	C7B—H71B	0.9700
C7A—H71A	0.9700	C7B—H72B	0.9700
C7A—H72A	0.9700	C8B—C9B	1.484 (3)
C8A—C9A	1.490 (3)	C8B—H81B	0.9700
C8A—H81A	0.9700	C8B—H82B	0.9700
C8A—H82A	0.9700	C9B—C10B	1.383 (4)
C9A—C10A	1.376 (3)	O2—H21	0.96 (3)
O1B—N1B	1.308 (3)	O2—H22	0.95 (4)
			
O1A—N1A—C2A	119.72 (18)	O1B—N1B—C9B	119.0 (2)
O1A—N1A—C9A	118.46 (17)	C2B—N1B—C9B	121.9 (2)
C2A—N1A—C9A	121.83 (19)	N1B—C2B—C3B	120.1 (2)
N1A—C2A—C3A	120.0 (2)	N1B—C2B—H2B	120.0
N1A—C2A—H2A	120.0	C3B—C2B—H2B	120.0
C3A—C2A—H2A	120.0	C2B—C3B—C4B	119.9 (3)
C2A—C3A—C4A	119.6 (2)	C2B—C3B—H3B	120.0
C2A—C3A—H3A	120.2	C4B—C3B—H3B	120.0
C4A—C3A—H3A	120.2	C3B—C4B—C10B	120.3 (3)
C3A—C4A—C10A	120.9 (2)	C3B—C4B—H4B	119.8
C3A—C4A—H4A	119.5	C10B—C4B—H4B	119.8
C10A—C4A—H4A	119.5	C10B—C5B—C6B	111.2 (3)
C6A—C5A—C10A	112.75 (19)	C10B—C5B—H51B	109.4
C6A—C5A—H51A	109.0	C6B—C5B—H51B	109.4
C10A—C5A—H51A	109.0	C10B—C5B—H52B	109.4
C6A—C5A—H52A	109.0	C6B—C5B—H52B	109.4
C10A—C5A—H52A	109.0	H51B—C5B—H52B	108.0
H51A—C5A—H52A	107.8	C7B—C6B—C5B	111.3 (3)
C7A—C6A—C5A	111.5 (2)	C7B—C6B—H61B	109.4
C7A—C6A—H61A	109.3	C5B—C6B—H61B	109.4
C5A—C6A—H61A	109.3	C7B—C6B—H62B	109.4
C7A—C6A—H62A	109.3	C5B—C6B—H62B	109.4
C5A—C6A—H62A	109.3	H61B—C6B—H62B	108.0
H61A—C6A—H62A	108.0	C6B—C7B—C8B	111.8 (2)
C6A—C7A—C8A	112.3 (2)	C6B—C7B—H71B	109.3
C6A—C7A—H71A	109.1	C8B—C7B—H71B	109.3
C8A—C7A—H71A	109.1	C6B—C7B—H72B	109.3
C6A—C7A—H72A	109.1	C8B—C7B—H72B	109.3
C8A—C7A—H72A	109.1	H71B—C7B—H72B	107.9
H71A—C7A—H72A	107.9	C9B—C8B—C7B	113.5 (3)
C9A—C8A—C7A	113.33 (19)	C9B—C8B—H81B	108.9
C9A—C8A—H81A	108.9	C7B—C8B—H81B	108.9
C7A—C8A—H81A	108.9	C9B—C8B—H82B	108.9
C9A—C8A—H82A	108.9	C7B—C8B—H82B	108.9
C7A—C8A—H82A	108.9	H81B—C8B—H82B	107.7
H81A—C8A—H82A	107.7	N1B—C9B—C10B	118.9 (2)
N1A—C9A—C10A	119.28 (19)	N1B—C9B—C8B	116.3 (2)
N1A—C9A—C8A	116.81 (18)	C10B—C9B—C8B	124.7 (2)
C10A—C9A—C8A	123.91 (19)	C9B—C10B—C4B	118.9 (2)
C9A—C10A—C4A	118.3 (2)	C9B—C10B—C5B	118.9 (3)
C9A—C10A—C5A	119.6 (2)	C4B—C10B—C5B	122.2 (3)
C4A—C10A—C5A	122.1 (2)	H21—O2—H22	102 (3)
O1B—N1B—C2B	119.10 (19)		
			
O1A—N1A—C2A—C3A	178.4 (2)	O1B—N1B—C2B—C3B	179.9 (2)
C9A—N1A—C2A—C3A	−2.0 (4)	C9B—N1B—C2B—C3B	−0.2 (3)
N1A—C2A—C3A—C4A	0.9 (4)	N1B—C2B—C3B—C4B	0.4 (4)
C2A—C3A—C4A—C10A	0.3 (4)	C2B—C3B—C4B—C10B	−0.5 (4)
C10A—C5A—C6A—C7A	49.6 (3)	C10B—C5B—C6B—C7B	52.8 (3)
C5A—C6A—C7A—C8A	−59.9 (3)	C5B—C6B—C7B—C8B	−61.4 (3)
C6A—C7A—C8A—C9A	38.5 (3)	C6B—C7B—C8B—C9B	37.8 (3)
O1A—N1A—C9A—C10A	−178.6 (2)	O1B—N1B—C9B—C10B	−179.94 (19)
C2A—N1A—C9A—C10A	1.7 (4)	C2B—N1B—C9B—C10B	0.1 (3)
O1A—N1A—C9A—C8A	0.5 (3)	O1B—N1B—C9B—C8B	−1.3 (3)
C2A—N1A—C9A—C8A	−179.2 (2)	C2B—N1B—C9B—C8B	178.68 (18)
C7A—C8A—C9A—N1A	172.3 (2)	C7B—C8B—C9B—N1B	174.06 (19)
C7A—C8A—C9A—C10A	−8.7 (4)	C7B—C8B—C9B—C10B	−7.4 (3)
N1A—C9A—C10A—C4A	−0.5 (4)	N1B—C9B—C10B—C4B	−0.2 (3)
C8A—C9A—C10A—C4A	−179.5 (2)	C8B—C9B—C10B—C4B	−178.7 (2)
N1A—C9A—C10A—C5A	178.5 (2)	N1B—C9B—C10B—C5B	178.77 (19)
C8A—C9A—C10A—C5A	−0.6 (4)	C8B—C9B—C10B—C5B	0.3 (3)
C3A—C4A—C10A—C9A	−0.5 (4)	C3B—C4B—C10B—C9B	0.4 (3)
C3A—C4A—C10A—C5A	−179.4 (3)	C3B—C4B—C10B—C5B	−178.5 (2)
C6A—C5A—C10A—C9A	−19.7 (4)	C6B—C5B—C10B—C9B	−22.2 (3)
C6A—C5A—C10A—C4A	159.2 (3)	C6B—C5B—C10B—C4B	156.8 (3)

### Hydrogen-bond geometry (Å, °)

**Table:**

*D*—H···*A*	*D*—H	H···*A*	*D*···*A*	*D*—H···*A*
O2—H21···O1A	0.96 (3)	1.87 (3)	2.825 (3)	170 (3)
O2—H22···O1B	0.95 (4)	1.86 (3)	2.799 (3)	170 (3)
C2B—H2B···O1A	0.93	2.53	3.454 (3)	171
C3A—H3A···O2^i^	0.93	2.50	3.392 (3)	160
C3B—H3B···O2^ii^	0.93	2.56	3.342 (4)	142
C5A—H52A···O1B^iii^	0.97	2.49	3.383 (4)	153

Symmetry codes: (i) −*x*+1/2, *y*+1/2, *z*; (ii) *x*−1/2, *y*, −*z*+1/2; (iii) −*x*+1/2, −*y*+1, *z*−1/2.
